# Psychiatric assessment in image-based sexual abuse case: a case report on imputability in personality disorder with narcissistic traits

**DOI:** 10.3389/fpsyt.2024.1395899

**Published:** 2025-01-22

**Authors:** Lorenzo Lodde, Claudia Zandara, Andrea Martella, Mirko Manchia, Pasquale Paribello, Irene Mascia, Martina Pinna

**Affiliations:** ^1^ Section of Psychiatry, Department of Medical Sciences and Public Health, University of Cagliari, Cagliari, Italy; ^2^ Forensic Psychiatry Unit, Sardinia Health Agency, Cagliari, Italy; ^3^ Department of Pharmacology, Dalhousie University, Halifax, NS, Canada

**Keywords:** forensic psychiatry, personality disorders, sexual abuse, imputability, cyber violence, case report

## Abstract

**Objectives:**

Crimes committed on the Internet and social networks are rising, and the phenomenon is complex. Knowledge of context would be useful for professionals in cases that need psychiatric assessment. We report on a case of a 27-year-old young adult who is accused of image-based sexual abuse and other crimes, for whom the examining judge requested psychiatric assessment.

**Methods:**

We conducted anamnestic collection (family, physiological and pathological, psychiatric, and toxicological), direct evaluation of the examinee, assessment of acquired health records, psychodiagnostic tests (i.e. graphic tests: Machover test, Koch test), Montreal Cognitive Assessment, Minnesota Multiphasic Personality Inventory 2, Pathological Narcissism Inventory, State-Trait Anger Expression Inventory 2, Toronto Alexithymia Scale, Thematic Apperception Test.

**Results:**

The clinical forensic assessment led to a diagnosis of Unspecified Personality Disorder (predominantly narcissistic traits) according to the DSM-5 criteria. Direct assessment showed a tendency to simulate or exaggerate symptoms, confirmed by the invalidation of the MMPI-2. In addition, the psychodiagnostic test showed a tendency to aggressive behavior and difficulty in identifying and describing emotions and feelings (alexithymia).

**Conclusions:**

This case highlights the importance of being familiar with the context of the Internet and social networks, where a rising number of crimes are committed. Forensic psychiatrists will be increasingly involved in evaluating cases related to the online world, which requires a basic knowledge of its characteristics and dynamics.

## 1 Introduction

In recent times, the advancement of technology and social networks has brought about some unpredictable consequences: alongside the advantages of connecting people and sharing information with unprecedented ease, there have also been unexpected and negative consequences of legal relevance.

The illicit dissemination of sexually explicit images or videos without consent is a phenomenon that violates the dignity, privacy and image of victims, exposing them to serious psychological, social and professional consequences. This practice, known as “revenge porn”, became a specific crime in Italy in 2019 with law no. 69/2019, which introduced article 612 ter. of the Italian criminal code.

Although new technologies are not illegal means per se and do not directly cause illegal behavior, web and social networks’ ease of use, alongside some of their distinctive features, make them facilitating means for criminal conduct, in particular with regard to sexual abuse and sharing digital material with sexually explicit content.

The first characteristic lies on the other side of the coin of the most intrinsically useful feature of the Internet and social networks: the ease of searching and finding information. Given that criminal conduct largely depends on the availability of information relevant to perpetrating crime, those means facilitate access and create more opportunities. The ease is not limited only to information: social networks, by their very nature, facilitate contact between individuals, making it easier to identify potential victims and monitor their online activity across the globe, effectively increasing the physically limited opportunities that can be found in the offline world (1).

A second feature to highlight is anonymity: in social networks, it is relatively easy to create an account without datalinks to oneself (a so-called “avatar” or “cyber alter-ego”), a factor which, by inducing a false sense of security, decreases social inhibition (2) and, in some cases, can facilitate illicit conduct. Contact on a social platform, often lacking direct face-to-face interaction, also allows rapid connection and “intimacy” through the projection of one’s own fantasies and needs onto a correspondent without specific characteristics, which are “filled” through imagination based on the available data, often just nickname and chat conversation. The possibility of creating a personal alter-ego could be attractive in subjects involved in marginalization and social stigma (including cases of mental distress or disorder), where online platforms offer a way of reintegration and acceptance in the online community through a fictitious identity, giving the subject a sense of belonging to a group. Groups that can act as catalysts for illicit conduct when there are conditions for suspension of individual responsibility (seen as shared) and positive reinforcement fostered by imitation: the so-called “herd behavior” (3). Furthermore, in an atmosphere of reciprocal incitement and risk underestimation, the sense of belonging acts as a driver for illicit conduct, and conversely, illicit conduct increases the sense of belonging. This dynamic can be particularly accentuated in subjects with an unstable or fragile self, associated with the need for admiration and validation to maintain a positive self-image.

Another cause for concern is material not extorted and shared without consent but generated through generative artificial intelligence. To date, A.I. is already capable of producing highly realistic images based on photo-video material present online, paving the way for the production of pornographic material (nude deep-fake, fakeporn) not only without the consent of the victims but without them even being aware of it.

Anonymity is also linked to a delicate topic in the online world: privacy. More and more tech companies proclaim it as their main value, trying to encourage the use of their digital platform by guaranteeing users complete protection of their digital personal data. However, the right to privacy necessarily clashes with other rights when crimes are committed. A powerful example is the “Apple - F.B.I. encryption dispute” case, where the company Apple Inc. did not agree to create software to unlock the device (an iPhone) of a terrorist involved in the San Bernardino massacre in 2015, presumably containing information relevant to the ongoing investigations (4). On this side, legislation in Europe is regulated by the GDPR (General Data Protection Regulation): the right to privacy is not applied in case of data processing carried out by authorities for investigation purposes (5).

Telegram represents a particular case among social networks: its features combine elements discussed above and have facilitated its use for illicit purposes. In particular, the platform allows users to send, receive and exchange documents of all types and formats (photos, videos, compressed attachments), as well as to create thematic channels (one-way sharing information from an account to participants) and groups (method of sharing information among members on an equal basis) through the use of invitation hyperlinks, shared between users or published on online sites. Groups can contain up to 200,000 participants, while thematic channels can theoretically reach an unlimited audience. In these chats, access is possible through administrator approval, and the user is guaranteed total anonymity by obscuring the mobile number, not sharing personal data, and using a nickname instead of a birth name (6). The permanence of the group depends on the user posting and sharing material relevant to the topic at hand, in this case, extorted pornographic material or revenge porn: this means that users, if not willing to share material deemed suitable by the group and its administrators, can be expelled (“banned”) from the chat (7). This factor incentivizes illicit conduct and triggers the psychological imitation mechanisms mentioned above. These characteristics make Telegram an effective means for disseminating illicit content and a potential risk for users who use it casually or unwarily, as they may incur criminal liability. In this context, understanding the psychological dynamics of offenders is crucial. Narcissistic traits, a desire for validation, and the anonymity afforded by online platforms could often be linked to these behaviors. The growing reliance on digital platforms for social interaction may exacerbate these traits, with offenders seeking power and control over others through manipulation and exploitation. Additionally, legal defenses based on mental health conditions complicate forensic assessments, especially when offenders claim diminished responsibility for their actions. These factors present unique challenges for forensic psychiatrists tasked with evaluating mental capacity in cases involving digital crimes. Knowledge of the use of online platforms and social networks for illicit purposes and, in this case, for sharing pornographic material without consent is relevant in the legal field and, in particular, for forensic psychiatrists, given the current increase in cases and the rapid evolution of the technologies in question, as well as the social and cultural changes that they bring about.

Finally, the global nature of digital platforms raises concerns about jurisdiction and the effectiveness of national laws in combating cross-border offenses. This highlights the need for international cooperation and the development of more robust privacy and data protection frameworks, as well as public education on responsible online behavior. Addressing image-based sexual abuse requires a multi-disciplinary approach that combines legal reform, technological innovation, and psychological support for both victims and offenders.

Here, we report a case of forensic psychiatric assessment carried out on a young adult accused of image-based sexual abuse.

## 2 Case description

Mr Z., a 27-year-old young adult, is accused of crimes committed between September 2020 and May 2022: illicit dissemination of sexually explicit images or videos (art. 612 ter c.2 of the Italian criminal code), private violence (art. 610 of the Italian criminal code), child pornography (art. 600 ter of the Italian criminal code), solicitation of minors (art. 609 indices of the Italian criminal code) and defamation (art. 595 of the Italian criminal code).

The examining magistrate requested a psychiatric report to evaluate the current defendant’s capacity to understand his acts and, at the time of the facts, to assess his current psychiatric social danger and his competence to stand trial.

We examined documents, anamnestic collection (family, physiological and pathological, psychiatric and toxicological), direct evaluation of the examinee with a psychiatric evaluation, the assessment of the acquired health documentation, psychodiagnostic tests (graphic tests: Machover test, Koch test); Montreal Cognitive Assessment; Minnesota Multiphasic Personality Inventory-2; Pathological Narcissism Inventory; State-Trait Anger Expression Inventory 2; Toronto Alexithymia Scale; Thematic Apperception Test.

No relevant antecedents emerged regarding physical health. Common childhood rashes were reported. In childhood, frequent hospital admissions were reported for unspecified recurrent upper airway problems. He did not suffer from any pathology, nor was he taking pharmacological therapy at the moment of assessment.

The examinee’s academic experience began positively during elementary school, where he exhibited good conduct and performed well academically. However, issues started to emerge during middle school, where his behavior became problematic, although his academic performance remained stable. As he entered secondary school, the pattern of poor conduct persisted, now accompanied by a significant decline in academic performance, culminating in several failures. These behavioral and academic challenges during his adolescence likely reflected deeper emotional and psychological struggles, which further impacted his ability to engage in the learning environment and form positive relationships with peers and authority figures.

On a psychopathological level, no psychiatric antecedents emerged. He reported the use of cannabinoids from the age of 12 in a social and, subsequently, family context, with an estimated quantity of around 10 g/day. He also reported the use of other substances, including the use of clonazepam since the age of 15, occasional methylenedioxymethamphetamine (MDMA) from the age of 16 for two years, and alcohol.

His parents had a highly conflicted marital relationship, marked by frequent disputes and a general lack of harmony. The stability of the family unit was further compromised by ongoing socio-economic difficulties and both parents’ substance abuse problems. These stressors created a volatile and unpredictable environment, which likely contributed to early emotional challenges. The lack of a supportive and secure familial foundation may have hindered the development of healthy attachment patterns, exacerbating the relational difficulties that would later emerge in interpersonal relationships.

The mother died by suicide when the examinee was 3 years old. Since then, he was raised by his father, described as violent and abusive. There are no psychiatric diagnoses or hospitalizations in acute inpatients unit in the family.

### 2.1 Timeline of the facts in question

In the period between 2019 and 2022, Mr Z. accessed numerous chat groups on the Telegram digital platform focusing on the sharing of pornographic and child pornography material. The chats also served as a context for online sexual encounters, allowing users to exchange contacts and initiate a private communication channel. Mr. Z. obtained the explicit photographic material of the victims through various online platforms, primarily by joining sexual-themed Telegram groups. Within these groups, he initiated private conversations with individuals, building trust and asking for explicit images of themselves. Once the victims shared these images, he would use them as leverage, threatening to expose the material unless the victims complied with further demands. This method of coercion was often accompanied by psychological manipulation and verbal violence, where Mr. Z. created an environment of fear and helplessness to ensure the victims’ submission. Among the users of the chats in question, there were also adolescents. In this context, Mr Z. would have carried out illicit conduct, including the solicitation of minors, private violence and child pornography through the use of the threat of online publication of material concerning the victims if they refused to send more pornographic material, as well as the publication of sensitive data (telephone number and address) of the victim on a dating site of a sexual nature and on a Telegram group of the same type. Two minor victims have reported to the Police, resulting in investigations and trial. During the investigation, Mr Z. spontaneously requested an evaluation at the psychiatric ward for referred psychomotor agitation and suicidal ideation following a home perquisition by the Police. He was offered hospitalization, but he refused, agreeing to take anxiolytic therapy (promazine) and to take the proposed pharmacological treatment at home (aripiprazole 10 mg/day),which he then admitted he had never taken. He was then discharged from the hospital.

### 2.2 Mental state examination

The examinee came for the assessment at the right time, showing a collaborative attitude. He appeared aware of the examiner’s role and fully oriented in space, time and persons. His affect appeared reactive, with an adequate range of facial expression changes. Throughout the interview, his speech was fluid and coherent, allowing for a reciprocal conversation. There was no formal thought disorder and no delusional content in his thinking, with some evidence of grandiosity, narcissistic in nature. His mood was reportedly euthymic, with psychological anxiety as a reaction to the present situation. His cognitive and intellectual functions were within the normal range for age and socio-cultural level. His volition also appeared preserved, as his instincts. He displayed good critical and judgmental skills. During the interview, the examinee often referred to the fact that, in his own opinion, his conduct was caused by a mental illness or alteration caused by the context (such as internet addiction or similar).

The results of the tests performed with the defendant are summarized in **Table 1**. We assessed cognitive skills with the Montreal Cognitive Assessment (8), which showed preserved cognitive functions. Specific personality tests were also employed to characterize the examinee, comprising 1) the Pathological Narcissism Inventory 52 (9), which highlighted the presence of a stable narcissistic core of the personality; 2) the Machover and Koch tests (10–14), a projective test which highlighted the grandiosity of the self, anxiety, and difficulty in adapting to the rules; 3) the Thematic Apperception Test (15, 16), an additional projective test which highlighted the control of the content that emerged through affectively neutral, morally conventional and stereotyped narratives and the absence of elements suggestive for the presence of psychotic disorders; 4) the Minnesota Multiphasic Personality Inventory-2 (17, 18), whose results were invalid due to simulation of disorders and poor adaptation; 5) the State-Trait Anger Expression Inventory-2 (19) which outlined the tendency towards aggressive verbal or physical behaviors that the personisable to control and inhibit; 6) the Toronto Alexithymia Scale-20 (20) positive for the presence of alexithymia.

**Table 1:** Psychodiagnostic tests scores.

Montreal Cognitive Assessment (MoCA):
1. Executive Visuo-spatial score 3/5;
2. Denomination 3/3;
3. Attention 2/2; Sustained attention 1/1; Calculation 2/3;
4. Language 2/2;
5. Fluency 1/1;
6. Abstraction1/2;
7. Deferred recall 4/5;
8. Orientation 6/6;
9. Total score 25/30.
Minnesota Multiphasic Personality Inventory 2 (MMPI-2):
1. VRIN T 49; TRIN T 57V;
2. Frequency scale – F T 111;
3. Scale F of the second half – Fb T 113;
4. Infrequent psychopathological scale – Fp T 97;
5. Lying scale – L T 39;
6. Defensiveness scale – K T 36;
7. Superlative self-representation scale – S T 33;
8. Simulation index – F – K T 27.
Pathological Narcissism Inventory (P.N.I. 52):
1. Total Mean 3.39 (M: 1.86; S.D.: 0.69);
2. Grandiose narcissism Mean 2.87 (M: 2.06; S.D.: 0.74);
3. Vulnerable narcissism Mean 3.77 (M: 1.77; S.D.: 0.72).
State-Trait Anger Expression Inventory-2 (STAXI-2):
1. State anger: T 72;
2. Feeling Anger: T 63;
3. Feeling how to express anger verbally: T 71;
4. Feeling how to express anger physically: T 74;
5. Trait anger: T 68;
6. Anger Temperament: T 88;
7. Anger Reaction: T 41;
8. Expression of anger externally: T 62;
9. Expression of anger internally: T 77;
10. External anger control: T 37;
11. Internal anger control: T 43;
12. Anger expression index: T 71.
Toronto Alexithymia Scale (TAS-20):
1. Difficulty identifying feelings: mean 3.57;
2. Difficulty describing feelings: mean 3.8;
3. Externally-oriented thinking: mean 3.25;
4. Total score: 73.

## 3 Discussion

Mr. Z. reported an existential story characterized by the loss of the main family reference figure at an early age, leading to a non-harmonious growth and development. The relationship with the remaining parental figure was reported as ambiguous and, at times, violent, probably leading to the development of a dismissing-avoidant attachment style (21–23). It could be inferred that such predisposition might have led, in turn, to prefer superficial and short relationships in an attempt to live one’s own emotional life in conditions of self-sufficiency through the development of a narcissistic personality, of an unattainable false self with grandiose thoughts, even if without delusional quality. This results in behaviors such as manipulation, predation and sexual exploitation, use of substances, gambling, excessive use of social networks, methods found in narcissistic personalities and implemented with the aim of feeling alive, adrenaline-filled and invincible (24, 25). The non-satisfaction of narcissistic needs creates a strong emotional vulnerability, the so-called “narcissistic wound” which brings with it a drop in hypertrophic self-esteem for even minimal wrongs, leading to a vision of oneself as a failure and ridiculous, amplified by the “stage” made up by the social network. This involves the implementation of behaviors underlying feelings of contempt, hatred and anger towards the other (and towards oneself), aimed at restoring the grandiosity of the self (26).

The picture that emerged leads to a diagnosis of Unspecified Personality Disorder according to the Diagnostic and Statistical Manual of Mental Disorders-5^th^ edition (with predominantly narcissistic traits) (27). The assessment results suggest that the defendant does not have a mental illness of significant severity in a forensic-psychiatric setting. He is, therefore, judged mentally capable and competent to stand trial.

Mr. Z. was found guilty of the charges brought against him. The sentence took into account mitigating circumstances and the reduction granted for choosing a summary trial. Additionally, he was permanently banned from holding any position in schools of any level, as well as from any role or service in public or private institutions regularly attended by minors.

By definition, personality traits develop in childhood and adolescence. As such, the reported narcissistic traits were present at the time when the crime was committed. However, according to the forensic psychiatric definition, the clinical severity of the reported narcissistic traits does not fall within the criteria for a personality disorder with a legally relevant value. In Italian law, for a personality disorder to be legally relevant in the mental capacity assessment and support the invocation of insanity defense, there must be evidence at the time of the event for functional manifestations of “diffusion of identity”, up to the “loss of identity” with dissociative or psychotic experiences and altered sense of reality, possibly culminating in the inability to differentiate self from non-self (28). During the interviews held with Mr. Z., he tried on several occasions to influence the examiner for an apparent secondary gain(i.e., the examinee claimed multiple times that a mental disorder supposedly caused his conduct). However, Mr. Z. always showed awareness of his actions and an adequate ability to recollect the memories surrounding the events, with a preserved sense of agency regarding his actions. He is, therefore, judged capable of understanding his acts at the time of the facts. Since Mr. Z. is not considered affected by a legally relevant mental disorder, according to Italian law, the social dangerousness cannot be determined. However, it appears advisable to rely on a mental health service for psychotherapeutic interventions that may promote building a greater degree of awareness of the reported psychological elements on the defendants’ part and how to manage them.

In addition to the developmental history outlined, it is important to consider the broader socio-cultural context in which Mr. Z. may have navigated his emotional and relational struggles. The constant comparison to idealized images and the pursuit of validation through online interactions may have exacerbated Mr. Z.’s tendency to rely on external sources for self-worth, further fueling his narcissistic needs. Moreover, the anonymity and instant gratification afforded by digital spaces could have reinforced maladaptive coping strategies, such as predatory behavior and manipulation, that he employed to gain control over others and fulfill an emotional void. These external influences, while not solely responsible for his condition, likely played a role in exacerbating the underlying personality traits and reinforcing dysfunctional behaviors.

Furthermore, while Mr. Z. demonstrated awareness of his actions during interviews, it is also worth considering the psychological mechanisms of defense he may have employed, such as denial or rationalization, which could obscure his ability to fully recognize the impact of his actions on others. These defense mechanisms are often prominent in individuals with narcissistic traits, as they strive to protect their fragile self-image from any form of criticism or vulnerability. Therefore, future therapeutic work should not only address his narcissistic tendencies but also explore the deeper emotional wounds that contribute to his self-perception and behavior.

Therapeutically, it is essential that any intervention focuses on the development of emotional regulation and empathy. Building greater self-awareness regarding the root causes of his narcissistic tendencies, along with exploring the defense mechanisms that perpetuate his emotional detachment, would be crucial in helping Mr. Z. develop more adaptive relational strategies. Psychotherapy should aim to help him confront the unresolved trauma from his early childhood and the ambiguous relationship with his remaining parental figure, as these experiences likely laid the foundation for his current behavioral patterns. Addressing these issues in a therapeutic context could help mitigate the risk of further harmful behavior and promote healthier ways of relating to others.

Finally, while Mr. Z. is not deemed legally insane under the provisions of Italian law, it remains important to monitor his psychological state over time. His narcissistic traits, while not legally relevant in this case, could contribute to future difficulties in interpersonal relationships and emotional regulation, and ongoing mental health support is advised. A comprehensive mental health evaluation and long-term psychotherapy may offer him the opportunity for meaningful change, potentially reducing the risk of future offenses and fostering a more balanced sense of self.

## 4 Conclusions

For a correct psychiatric evaluation, and even more so in the forensic-psychiatric field, it appears useful, if not necessary, to know the context in which the actions and the facts under examination take place. With the growing popularity and use of the Internet and social networks for criminal purposes, forensic psychiatrists will be increasingly involved in evaluating cases related to the online world, which requires basic knowledge of its characteristics and dynamics. The specific understanding of how these platforms are used for illicit purposes allows the correct classification of the individual in the environment where they find themselves and the motivations, meanings and forces that push them to act. Being informed about what represents the “norm” in a certain context allows the examiner to detect the adequacy of behavior from a clinical point of view. This case is to provide information about the evaluation of a subject not suffering from a major mental disorder and capable of using the minor victims’ ignorance, immaturity and vulnerability to his advantage, a situation in which the former’s knowledge played an important role.

## Data Availability

The original contributions presented in the study are included in the article/supplementary material. Further inquiries can be directed to the corresponding authors.
